# A study of new *NEK8 *mutations in patients with severe renal cystic hypodysplasia and ciliopathy-associated defects

**DOI:** 10.1186/2046-2530-4-S1-P54

**Published:** 2015-07-13

**Authors:** V Grampa, M Delous, F Silbermann, G Oyde, P Krug, E Filhol, JL Alessandri, S Sigaudy, R Bouvier, MT Zabot, C Antignac, M Gubler, T Attié-Bitach, A Benmerah, C Jeanpierre, S Saunier

**Affiliations:** 1 INSERM U1163, Institut IMAGINE Laboratory of Inherited Kidney Diseases, Paris, France; 2 INSERM U781, Institut IMAGINE, Hôpital Necker-Enfants Malades, Paris, France; 3 Paris Descartes-Sorbonne Paris Cité University, Institut IMAGINE, Paris, France; 4 Réanimation Néonatal et Infantile, Centre Hopitalier Felix Guyon, St. Denis, Réunion, France; 5 Département de Génétique Médicale, Hopital de la Timone, Marseille, France; 6 Centre de Biologie et de Pathologie Est, Bron, France; 7 APHP - Département de Génétique, Hôpital Necker-Enfants Malades, Paris, France

## 

*NEK8/NPHP9 *encodes a NIMA (Never-In-Mitosis A) protein essential for cell cycle control. NEK8 is composed of kinase and RCC1 domains, the latter involved in centrosomal localization. It localizes into the nucleus and at the inversin compartment in the primary cilium. Using ciliary gene-enriched exome sequencing, we identified recessive *NEK8 *mutations in 3 cases with severe overlapping phenotypes including renal cystic (hypo)dysplasia, *situs inversus*, cardiopathy and paucity of bile ducts. Two patients who died early after birth carried missense mutations in the kinase and/or RCC1 domains. A homozygous splice mutation was identified in a fetus with Meckel-like phenotype. Analyses of patient fibroblasts and IMCD3 cells expressing mutated NEK8-GFP revealed that the mutations affect NEK8 nuclear and ciliary localization. The number of ciliated cells was reduced and ciliary localization of NEK8 partner ANKS6/NPHP16 was lost, demonstrating the key role of NEK8 in cilia function. Surprisingly, in patient fibroblasts, NEK8 accumulates at the Golgi that appeared dispersed into the cytoplasm suggesting a role in vesicular trafficking. Cell cycle defects associated with abnormal nuclear accumulation of YAP, a transcriptional co-activator of the Hippo pathway was also observed, together with dysregulation of several Hippo effector/target genes. Finally, injection of *nek8 *morpholinos in zebrafish embryos led to ciliopathy-related phenotype (curly body axis, laterality defects, pronephric cysts) that could be rescued by RNA expression of WT NEK8 but not by the mutated forms, further demonstrating pathogenicity of the mutations. Altogether, we demonstrate that human *NEK8 *mutations alter developmental ciliary and non-ciliary processes, thus leading to multisystemic defects.

